# Microbial and Chemical Characteristics of Doogh (Iranian Fermented Milk Drink)

**DOI:** 10.1155/2021/3009795

**Published:** 2021-12-17

**Authors:** Sara Mohamadi, Vahid Mofid, Tayebeh Zeinali, Anosheh Rahmani, Parisa Sadighara, Leila Peivasteh-Roudsari

**Affiliations:** ^1^Department of Food Hygiene and Quality Control, Faculty of Veterinary Medicine, Shahrekord University, Shahrekord, Iran; ^2^Department of Food Science and Technology, Faculty of Nutrition Science & Food Technology, National Nutrition and Food Technology Research Institute, Shahid Beheshti University of Medical Sciences, Tehran, Iran; ^3^Social Determinants of Health Research Center, Department of Public Health, Faculty of Health, Birjand University of Medical Sciences, Birjand, Iran; ^4^Department of Food, Halal and Agricultural Products, Food Technology and Agricultural Products Research Center, Standard Research Institute (SRI), Karaj, Iran; ^5^Department of Environmental Health, Food Safety Division, School of Public Health, Tehran University of Medical Sciences, Tehran, Iran

## Abstract

**Background:**

Regarding the increasing public health concerns about the safety of foodstuffs, the current survey was designed to argue the presence of preservatives (e.g., sodium benzoate (SB), potassium sorbate (PS), and natamycin) and also the level of salt and fungi in 148 samples of yoghurt drink “Doogh.”

**Methods:**

The enumeration of fungi and determination of salt content of samples were performed according to the standard procedures. Preservative determination was performed by reverse phase high-performance liquid chromatography with UV detection (RV-HPLC-UV).

**Results:**

0.1% of the total analyzed samples was above the permitted level of Iranian standard for SB (0%), while PS was not detected in any of them. Furthermore, natamycin in 0.11% of the analyzed samples had more than the permissible level of Iranian standard. Additionally, about 15% of the tested samples was higher than the Iranian standard level for fungi (<102 CFU/mL). The average amount of salt in the tested Doogh samples and also in the examined Kefir samples was significantly (*P* < 0.001) lower than the standard amount of salt (<0.8 g/100).

**Conclusion:**

In conclusion, the quality of Doogh and Kefir samples were acceptable in terms of salt content. Kefir had a significantly (*P* ≤ 0.001) lower amount of salt in comparison with Doogh. Taken together, underlining the results of the present study, no significant public health concern would exist respecting the mentioned additives.

## 1. Introduction

Nowadays, due to increasing public awareness about the harmful consumption of carbonated beverages, desires to use the natural drinks such as Kefir and Doogh are rising [1, 2]. Kefir is a fermented probiotic milk drink, which is considered as a powerful dietary supplement. It is produced from the incubation of Kefir grains with water or raw milk and originated from the Balkan–Caucasian region. Actually, Kefir grains are considered to be symbiotic associations with a mixture of yeast and bacteria. The major components of Kefir are lactic acid, ethanol, and CO_2_. It is confirmed to have multiple health beneficial features like antifungal, antibacterial, antiallergic, antioxidant, anti-inflammatory, and antitumor effects [2]. Doogh is another fermented Iranian dairy drink produced from adding salt (maximum 1%), water (50–60%), and also, an essence of aromatic vegetables (e.g., thyme, mint, and oregano) [3] to set yoghurt (40–50%) [4–6], which is finally acidified (pH < 4.5) by fermentative bacteria including *Streptococcus thermophilus* and *Lactobacillus delbrueckii* ssp. *Bulgaricus* [5, 7]. It is also known with other titles such as “*Ayran*” in Turkey and “Lassi” in India [4]. Doogh has turned to the most favorite traditional dairy drink in the Middle East [6, 7]. Considering its probiotic bacterial content, high value of nutrients and also several health-promoting potentials (i.e., ameliorating lactose digestion, agitating the immune system, and lowering serum cholesterol levels) [8], as well as pleasant organoleptic properties, this fermented product is also a unique source of high-quality proteins, phosphorus, calcium, magnesium, zinc, and B vitamins like riboflavin (B_2_), niacin (B_3_), pyridoxine (B_6_), and cobalamin (B_12_). From nutritional point of view, Doogh includes all the compounds existing in yoghurt. Moreover, higher digestibility, calcium uptake, vitamins, and nutritional metabolites in comparison with milk have made Doogh a popular and nutritional product. Additionally, Doogh consumption in each meal would decrease the number of pathogenic bacteria significantly and can prevent microbial contamination [9].

High level of nutrients as well as low pH has made Doogh an ideal media for the proliferation of molds and yeasts [7]. Thus, microbial putrefaction referable to fungi describes as a serious complication for dairy industries, because of their capability to survive at low temperatures and acidic pH [10]. Different factors like the initial microbial quality of raw milk, inadequate thermal processing of milk, used starter cultures, and secondary microbial contaminations via equipment, air, ventilation, and packaging material have alter the quality and shelf life of the final product which in turn lead to the spoilage of Doogh by acid-resistant microorganisms, especially yeasts, molds, and bacteria during storage and safety reduction of this product [4, 6, 11, 12]. Fungal spoilage of Doogh is not only related to its consumers' acceptability and as a result of economic losses but also the growth of specific fungi can induce the production of mycotoxins, which are known to be severely harmful to humans [10].

For this purpose, the antimicrobial preservatives are usually added to Doogh in order to stop or delay unwanted microbiological, chemical, or enzymatic alterations and enhancing their shelf life. They also prohibit hazards for users due to the existence of microbial toxin or pathogenic microorganisms. However, the unpleasant effects of these additives have been reported in some surveys [4, 11–14]. Typically, sodium benzoate (SB), potassium sorbate (PS), and natamycin are the most regularly used preservatives in Doogh as antifungal agents [11, 13]. Although SB, PS, and natamycin are generally recognized as safe (GRAS), nevertheless, the incidence of allergic reactions to SB in humans, like Uriticaria, nonimmunological contact Urticaria, convulsions, weak clastogenic activity, metabolic acidosis, hyperpnoea, and asthma, has been announced in some studies [8, 15–18]. Further investigations revealed that PS has a comparatively lower toxicity to humans, since it is promptly metabolized in the same pathway as the other fatty acids. In humans, a few cases of idiosyncratic intolerance to PS have been reported [8, 15, 16].

In Iran, Food and Drug Organization (FDO) and Institute of Standards and Industrial Research of Iran (ISIRI) have forbidden these additives in the dairy products [4, 11, 12] and determined all physical, chemical, physicochemical, microbiological, and sensory characteristics of Doogh (ISIRI: No. 2453 2008; ISIRI: No. 761 2012; ISIRI: No. 14345 2012) [15, 19]. Despite the ban on preservative use in Doogh, some dairy manufacturers may use different preservatives illegally in order to solve the microbial contamination, particularly in summer [11, 12]. So, surveillance of these preservatives is crucial not only for food safety and quality assurance purposes but also for human safety [15, 18].

Taking these into consideration, the objective of the current research was to quantify the mentioned preservatives, salt content, and fungi level in commercial bottled Dooghs and kefir in different cities of Iran.

## 2. Methods and Materials

### 2.1. Sampling

In the present study, 60 samples from different brands of Iranian commercial Doogh and 88 samples of Kefir drinks were purchased randomly from local vendors located in various cities of Iran, from the 6^th^ of January until the 14^th^ of March 2021. The sampling was performed according to the standard and national procedures (ISIRI, 326). All samples were stored below 4°C before analysis. Doogh sample was examined for the three preservatives (SB, PS, and natamycin), salt content, and fungi count, while Kefir samples was solely tested for salt level.

#### 2.1.1. Standards and Reagents

High-purity commercial standards of SB, PS, and natamycin were purchased from Sigma-Aldrich (Wisconsin, USA). Other reagents of HPLC such as methanol, acetonitrile, ammonium acetate, glacial acetic acid, chloridric acid, and petroleum benzene were obtained from Merck (Darmstradt, Germany). Deionized water was prepared through the Millipore Milli-Q water purification system (ELGA, UHQ-II-MK3, and UK).

#### 2.1.2. Reverse Phase High-Performance Ultraviolet (RV-HPLC-UV)

The RV-HPLC-UV techniques were applied to measure preservatives in Doogh samples in accordance with the procedure described by Esfandiari et al. (2013) [11]. A 10 mL of homogenized Doogh was accurately transferred into a 25 mL volumetric flask and diluted with mixture of buffer acetate 0.1 M (pH = 4.5) : methanol (2 : 1, *v*/*v*). The solution was treated by ultrasonication, centrifuged in 5000 rpm, both for about 10 min. The mixtures were then filtered through a 0.45 *μ*m polytetrafluoroethylene (PTFE) membranous filters, and 20 *μ*L was injected into the HPLC by auto sampler. Chromatographic analysis was performed on 1200 series HPLC from Agilent technology equipped with automated sample injector, C18 analytical column (250 × 4.6 mm, 5 *μ*m), ultraviolet detector, high-pressure pump, and degasser. The separation was carried out with a flow rate of 0.8 mL/min, and the UV detector was set at 225, 250, and 303 nm for SB, PS, and natamycin, respectively. The peaks were diagnosed based on the retention time. The data was processed in the Millennium 32 software. Correlation coefficient value was 0.996 for all the preservatives.

### 2.2. Determination of Salt Amount

The unionized salt content in Doogh and Kefir samples were tested based on the standard procedures (ISIRI, 26).

### 2.3. Determination of Fungal Colony Count

The enumeration of colony-forming units of yeasts and/or molds of Doogh samples was performed at 25°C according to the standard and national procedures (ISIRI, 10154) and the following equation:

(1) $$\begin{array}{c} N=\frac{\sum C}{\left(n1+0.1n2\right)d}, \end{array}$$

∑*C*: the total number of colonies which counted on two chosen plate from 2 serial dilutions

*n*_1_: the number of plates with maximum of 150 and minimum of 10 colons in the first countable dilution

*n*_2_: the number of plates with maximum of 150 and minimum of 10 colons in the second countable dilution

*d*: dilution coefficient in the first selected dilution.

### 2.4. Statistical Analysis

*T*-test in SPSS software (version 16) (SPSS Inc., Chicago, IL, USA) was utilized to analyze the difference of distribution between the mean concentration of salt levels of Doogh and Kefir samples. The results were expressed as mean ± SEM. Statistical significance was set at *P* < 0.01.

## 3. Result and Discussion

### 3.1. Preservatives (SB, PS, and Natamycin)

The percentage of preservatives, molds, and yeast content of positive Doogh samples is shown in Table 1. In the current research, SB (<10 mg/L) was only detected in 0.1% of the total analyzed samples which ranged from 3.9 to 7.1 mg/L that is above the permitted level of Iranian standard, while PS was not detected in any of them. Natamycin was detected in 0.11% of the analyzed samples ranged from 1.22 to 4.1 mg/L, which were more than the permissible level of Iranian standard (0%). The importance of food preservatives to consumers has always been a health security matter [7, 17]. SB (C_7_H_5_NaO_2_) and PS (C_6_H_7_KO_2_) are the sodium salt of benzoic acid and sorbic acid, respectively [17]. The antimicrobial activity of PS is attributed to conjugated double bonds and reactive carboxyl group in its structure. The combination of SB and PS are more effective than their individual counterpart [20]. SB and PS have different antibacterial effects that are dependent on pH level. SB, as an antibacterial agent, is mostly used for foodstuffs with pH < 4.5, whereas PS, as an antifungal agent, is used for food products with pH < 6.5 [16, 17]. Like other weak acids that prevent the growth of microbes, they are more active in low pH and incapable at neutral pH. This indicates that these acids have antimicrobial effect on not ionized format, because the lipophilic undissociated molecule is easily permeable throughout the cell membrane and appears its destructive effects on the cells [8, 18, 20]. Sorbic acid is a polyunsaturated fatty acid (PUFA); thus, its metabolism in humans as well as other acids generally shows less toxic effect than benzoic acid [1]. Natamycin (C_33_H_47_NO_13_) also known as pimaricin is a polyene macrolide with antifungal effects which was generated by aerobic fermentation of Streptomyces natalensis and related species [11, 19, 21], but inactive against bacteria [22]. It is a white, odorless, and tasteless powder [21] which is considered to be safe in food industry due to its sensitivity to ultraviolet light and acidic pH [22]. Its low solubility in water and most organic solvents makes it appropriate for the surface treatment of foods like cheese and Doogh [11, 19].

The use of chemical additives in various food products is well regulated by several safety organizations worldwide [11]. The US Food and Drug Administration (FDA) declared these preservatives as GRAS which can be used in foods on the provision that should be declared on the label, and also, its usage should not be more than maximum permitted level (MPL) (i.e., SB < 0.1%, PS < 0.2%) [15, 17, 18]. The MPL of natamycin in yoghurt in the USA is 5-10 mg/L [22]. In the European Union, the MPL of SB and PS in dairy-based drinks is 150 and 300 mg/kg, respectively (European Commission 2008) [15]. Moreover, the Joint FAO/WHO Expert Committee on Food Additives (JECFA 1973) evaluated this additives several times and found them to be acceptable for use in foods [23], because the acceptable daily intake (ADI) values have been reported as 0–5 mg/kg body weight/day for SB, 0–25 mg/kg body weight/day for PS, and 0-0.3 mg/kg body weight/day for natamycin [1, 11, 18, 23]. But its use is prohibited in dairy products by Iranian National Standards.

Uncontrolled use of chemical food preservatives would lead to several health side effects such as allergic reactions (urticarial) and hyperactivity in children exposed to SB specially when consumed with food colorants [15, 23], although SB and PS have a very low mammalian toxicity [24, 25]. Yet, some genotoxicity effects, including the chromosome oddities in Chinese hamster cells, human lymphocytes, bone marrow cells of mice, and liver cell of pregnant rats have been illustrated for SB and/or PS. Therefore, pregnant women are recommended to avoid foods containing SB as an additive. Furthermore, scientists has declared that PS interaction with nitrite could produce genotoxic and cell-transforming agents, for instance, 1, and 4-dinitro-2-methylpyrrole and ethylnitrolic acid under situations of acidic pH, heating, and storage. Mamur et al. (2012) indicated that SB, at the highest concentrations (800 *μ*g/mL) in vitro, is genotoxic to the human peripheral blood lymphocytes [26]. However, natamycin appeared with a margin of safety greater than the aforementioned chemicals. Natamycin does not have any toxic, mutagenic, tetratogenic, and allergenic effect. In spite of mentioned hazardous effect of preservatives on human health, their safe usage would make the products shelf life expanded by reduction the rate of common microbial spoilage [11].

Similarly, Esfandiari et al. (2013) used RV-HPLC-UV to determine the amount of several preservatives (SB, PS, and natamycin) in the pasteurized Doogh in Iran. They concluded that among 39 studied samples, SB was observed in all the samples, while PS was not detected in any of them, and natamycin was detected in 10-25% of the samples [11]. The presence of SB in our study was the near agreement with Esfandiari et al. (2016) findings which ranged from 0.8 to 4.7 mg/L [4]. Yildiz et al. (2012) also found that the content of SB in Ayran samples in Turkey ranges from 1.54 to 16.57 mg/L. In another studies, Akbari-adergani et al. (2013) pointed out that all of the 27 Doogh samples in Iran contained SB ranged from 18.3 to 2345.1 mg/kg, and 25.9% of samples contained PS between 0.6 and 4961.3 mg/kg [27]. Additionally, Amirpour et al. (2015) applied HPLC method for analyzing the levels of SB and PS in 400 food samples, including 40 Doogh samples. They reported that all samples of Doogh included SB with the mean content of 20.8 ± 3.0 (ranged from 14.9 to 30.0 mg/kg), while the mean level of PS was 28.95 mg/kg [15]. In similar study in Iran, Zamani Mazdeh et al. (2014) explained that all 130 Doogh samples contained SB ranging from 14 to 30 mg/kg, whereas PS was detected in only 13% of them with the average content of 13.3 ± 39.6 mg kg/L; however, their usage on the product labels was not declared [14]. Another study was done by Bahremand et al. (2013) in Tehran considering that all of the 27 Doogh samples contained SB which ranged from 18.3 to 2345.16 mg/kg, while only 25.92% of all samples contained PS in range of 0-4961.3 mg/kg. And 25.9% of all samples contained both of SB and PS [8]. Additional survey was performed by Rahimirad. (2014) in 172 Doogh samples in West Azerbaijan, Iran, during 2011 to 2014; the prevalence of PS was reported as 14.6% of samples with the highest and the average concentration of 722.4 and 27.136 mg/kg, respectively [1]. Javanmardi et al. (2015) also demonstrated the occurrence of SB and PS in 60 different food samples including Doogh using HPLC. They declared that all 15 samples of Dooghs contained SB with a mean concentration of 242 ± 14.5 *μ*g/mL, while 20% of the samples contained PS with a mean level of 67 ± 4.7 *μ*g/mL [16]. Salehi et al. 2017 assumed that SB and PS were not found in any of 45 Doogh samples in Hamedan province of Iran using RP-HPLC [18]. Furthermore, the findings of Amirpour et al. (2015) survey indicated that SB can naturally produce in fermented dairy products like Doogh. For this reason, it would be reasonable to delineate a natural level, above which addition could be ascertained [15]. In this regard, the source of SB in Doogh can be attributed to the addition of herbal essence which contains benzaldehyde that produces benzoate and also autooxidation of benzaldehyde with specific strains of lactic acid bacteria. Moreover, using sodium chloride in Doogh may change benzoic acid into SB [15]. Benzoic acid also originates from the degradation of phenylalanine [16]. Additionally, hippuric acid which is naturally present in milk can consequently convert to benzoic acid by lactic acid bacteria [15]. But the point is that the benzoic acid content that originated from hippuric acid is very low; the highest concentration of hippuric acid in the milk was about 50 mg/kg [16]. As far as Doogh is a diluted and salted formulation prepared from yoghurt, processing yoghurt to Doogh leads to a decrease in the benzoic acid content with amount of around 60-75%. Accordingly, Esfandiari et al. (2016) hypothesized that the amount of 6 mg/L can be defined for benzoic acid as admissible limit for Doogh in Iran by Iranian supervision authorities [4]. In conclusion, a minimal level for SB in dairy products should be determined in the legislation [20]. Utterly, the reason for the diversity in the level of preservatives in Doogh samples in different studies is not evident but it may be associated to several factors like milk-producing animal feed, milking season, breeding conditions, hippuric acid level in raw milk, samples size, type of commercial lactic acid bacteria starter, processing technique, storage condition, and type of Doogh tested [4].

### 3.2. Fungi (Yeast and Molds)

In current research, the amount of fungi in 15% of the tested samples was higher than the Iranian standard level (<10^2^  CFU/mL). As far as Doogh and other fermented dairy products provides a selective condition for the growth of yeasts and molds due to their low pH, fungi are known as a major cause of spoilage of dairy products with side effects like CO_2_ and acid production [10, 12]. The most relevant fungi in Doogh are *Kluyveromyces lactis*, *Saccharomyces cerevisiae*, and *Geotrichum* sp. [10]. Besides economic losses, shelf life, and sensory property reduction of the final product, mycotoxins as fungal by-products pose a crucial potential risks to consumer health [1, 14]. Mycotoxins could cause carcinogenicity, malformations, and growth retardation, as well as immune systems suppression and even mutagenesis [28]. In this regard, Hatamkia et al. 2016 demonstrated that out of 200 samples, 56 (28%) were unqualified as a result of mold and yeast counts more than the maximum acceptable level defined by national standard of Iran (≤100 CFU/mL). It has been indicated that traditional dairy products are often contaminated more than industrial ones. It is probably due to different aspects which affect hygienic quality of Doogh such as starter culture, chemical compositions, added ingredients, and packaging materials [28]. This was illustrated by Mihyar and Yaman (1997), in Lebanese yoghurt products which were produced under absolute hygienic situations; thus, initial numbers of yeasts and relative concentrations of SB and PS were lowered [29].

### 3.3. Salt

The average amount (mean value) of salt in the tested Doogh samples (0.76 ± 0.01 mg/L) and also in the examined Kefir samples (0.44 ± 0.01 mg/L) was significantly (*P* < 0.001) lower than the standard amount of salt (0.8 g/100). The mean values of salt content of Doogh samples compared to Kefir samples and also their comparison with their Iranian standard levels are shown in Table 2. In general, the quality of Doogh and Kefir samples was acceptable in terms of salt content. There was a highly significant (*P* < 0.001) difference between the average amount of Kefir salt and the average amount of Doogh salt. Actually, Kefir had a significantly (*P* < 0.001) lower amount of salt in comparison with Doogh. Therefore, it is a better option for consumption by high-risk groups such as high blood pressure.

Based on regulations (ISIRI: No. 26), NaCl (<0.8 g/100) is used as another GRAS preservative in Doogh and Kefir [15]. Salt is essential for ordinary body function and food conservation. Nonetheless, abundant daily salt intake leads to illnesses such as cardiovascular disorders, high blood pressure, and gastric cancers. For instance, sodium intake of 3480 mg/day, identical to 8.8 g/day of salt, was the major cause of cardiometabolic illnesses, chiefly in elderly people, in the USA, as well as Iran in 2016 (9.52 g). According to the clinical negative effects, WHO has recommended a maximum salt consumption of 5 g/day. In this regard, the Iranian regulatory authorities have yielded new restrictions, as far as Iranian salt intake was almost twice the WHO recommended amount [13].

## 4. Conclusion

Taken together, underlining the results of the present study, no serious public health concern would arise relating to the argued preservatives. Therefore, in order to produce high-quality products, applying Hazard Analysis and Critical Control Point (HACCP), Good Manufacturing Practice (GMP), and Good Hygienic Practice (GHP) techniques is necessary. Additionally, comprehensive supervision of the dairy industry by legislation organizations to restrict uncontrolled usage of these preservatives is crucial, to assure human safety and quality control of products.

## Figures and Tables

**Table 1:** The frequency of positive Doogh samples (%).

Standard level	Sodium benzoate (0 mg/L)	Potassium sorbate (0 mg/L)	Natamycin (0 mg/L)	Mold/yeast (<10^2^ CFU/mL)
Positive items (%)	0.1%	0%	0.11%	15%
Range	3.9-7.1	0	1.22-4.1	2.4 × 10^2^ –1 × 10^5^

**Table 2:** Mean and concentration range of salt in Doogh and Kefir samples.

Group	Number of samples	Min (mg/L)	Max (mg/L)	Mean (mg/L) + SEM	Significance level with each other	Significance level with Iranian standard levels (<0.8 g/100 mL)
Doogh	60	0.5	0.94	0.76 ± 0.01	0.001	0.001
Kefir	88	0.04	0.91	0.44 ± 0.01	0.001	0.001

## Data Availability

Data is available upon reasonable request from the corresponding author.
